# Comparative study between biliary covered self-expandable metal stent and conventional endoscopic bile drainage treatment in endoscopic retrograde cholangiopancreatography-related Stapfer type II retroperitoneal perforations

**DOI:** 10.1371/journal.pone.0300029

**Published:** 2024-03-12

**Authors:** Jun Heo, Min Kyu Jung, Jieun Lee, Dong wook Lee, Chang Min Cho, Jung Guen Cha

**Affiliations:** 1 School of Medicine, Kyungpook National University, Daegu, The Republic of Korea; 2 Department of Internal Medicine, Kyungpook National University Hospital, Daegu, Republic of Korea; 3 Department of Internal Medicine, Kyungpook National University Chilgok Hospital, Daegu, Republic of Korea; 4 Department of Radiology, Kyungpook National University Hospital, Daegu, Republic of Korea; Morgagni-Pierantoni Hospital, ITALY

## Abstract

**Background:**

Perforation is one of the most serious complications of endoscopic retrograde cholangiopancreatography (ERCP). Conventional nonsurgical endoscopic treatments including intravenous antibiotic administration and plastic endoscopic biliary drainage are generally approved for the treatment of ERCP-related Stapfer type II perforation (perivaterian type). Biliary covered metal stent placement has recently been reported to have favorable outcomes in ERCP-related Stapfer type II perforations. We aimed to compare the outcomes of conventional endoscopic bile drainage and biliary covered self-expandable metal stent (SEMS) insertion in patients with Stapfer type II perforation.

**Methods:**

Medical records of patients who underwent ERCP at Kyungpook National University Hospital in Daegu from 2011 to 2022 were retrospectively reviewed.

**Results:**

A total of 8,402 ERCP procedures were performed in our hospital. Sixty-six ERCP-related perforations (0.78%) were identified. Among them, 37 patients (56.1%) who had Stapfer type II perforations were enrolled. Thirteen and twenty-four patients received biliary covered SEMS insertion and conventional endoscopic bile drainage treatments, respectively. No significant differences were observed in the clinical success rate (92.3% vs. 91.7%, p = 1.000), hospital stay (9.46 ± 5.97 vs. 13.9 ± 13.2 days, p = 0.258), and post-ERCP–related fasting time (5.4 ± 3.4 vs 4.3 ± 3.0 days, p = 0.305). Complications including bleeding, post-ERCP pancreatitis, fever, and death were not significantly different between the two groups. The conventional endoscopic bile drainage group took less time for ERCP than the SEMS group (11.5 ± 5.2 vs. 18.5 ± 11.2 min, p = 0.013).

**Conclusions:**

Compared with the conventional endoscopic bile drainage treatment method, biliary covered SEMS did not improve patient outcomes in ERCP-related Stapfer type II perforations.

## Introduction

Endoscopic retrograde cholangiopancreatography (ERCP) is one of the most effective treatment options for pancreaticobiliary disease. However, during ERCP, complications including perforation, bleeding, and pancreatitis may occur. Among the complications, the incidence of perforation ranges from 0.3% to 1.0% [1, 2]. When perforation occurs, as bile could leak through the perforated site into the retroperitoneum, immediate and appropriate management is significant. This process induces peritonitis and a septic condition, thereby leading to poor clinical outcomes. The mortality rate from post-ERCP procedure perforation is approximately 8%–23% [2, 3].

Post-ERCP–related perforations can be divided into the following four categories according to the Stapfer classification [1]: duodenal perforation of the lateral or medial wall, type I; perivaterian perforation, type II; ductal perforation, type III; and retroperitoneal air alone, type IV. Of the four perforation types, type II is the most common type and accounts for 15%–55% of ERCP-related perforations [1, 2].

The management of ERCP-related perforations can be divided into surgical and nonsurgical treatments. Conventional nonsurgical treatment includes nothing by mouth (nil per os, NPO), intravenous broad-spectrum antibiotic administration, and conventional endoscopic bile drainages. Conventional bile drainage methods include percutaneous drainage (e.g., percutaneous transhepatic biliary drainage [PTBD] and percutaneous transhepatic gallbladder drainage [PTGBD]) and endoscopic management using endoscopic nasobiliary drainage (ENBD) or plastic endoscopic retrograde biliary drainage (ERBD) stents. Recently, biliary fully covered self-expandable metal stents (SEMSs) have been reported to show favorable results in some Stapfer type II perforations [3, 4]. However, these studies had limitations of including ERCP failure cases and previously endoscopic sphincterotomy cases. We intend to understand the real clinical efficacy of the ERCP perforation treatment modality in cases with naïve duodenal papilla. Therefore, this study aimed to compare the efficacy of biliary covered SEMS and conventional endoscopic bile drainage in ERCP-related Stapfer type II perforations.

## Materials and methods

### Study design

Medical records of patients who underwent ERCP at Kyungpook National University Hospital in Daegu from 2011 to 2022 were retrospectively reviewed. Post-ERCP–related perforation was identified using fluoroscopy during the ERCP procedure or simple abdominal X-ray follow-up within 24 h following ERCP procedures. This study was approved by the Institutional Review Board (IRB) (approved number: KNUH 2020-09-011) and was conducted in accordance with the guidelines of the Declaration of Helsinki (1989 revision). Data were accessed for research purposes from December 1, 2020 to May 31, 2023. All data were fully anonymized and the IRB waived the requirement for informed consent.

### Patients

Data from patients who underwent ERCP at Kyungpook National University Hospital were analyzed. The following were the inclusion criteria: (1) definite or suspicious perforation was described in ERCP reports, (2) when retroperitoneal air was confirmed during ERCP or immediately following ERCP fluoroscopy or simple abdominal X-ray or other imaging modalities, (3) ERCP-related Stapfer type II perforation (perivaterian), and (4) endoscopic bile drainage (e.g., ENBD, plastic ERBD, and biliary covered SEMS insertion) was performed for the treatment of ERCP-related perforation. The following were the exclusion criteria: (1) ERCP was performed using a papillectomy procedure (e.g., ampullary adenoma), (2) history of ERCP sphincterotomy or ERCP/PTBD transpapillary balloon dilatation, and (3) ERCP-related Stapfer type I/III/IV perforations.

### ERCP procedures

ERCP procedures were performed for in-hospital patients with close monitoring and follow-up. All ERCP procedures were performed by two experienced pancreatobiliary specialists who had experienced more than 3,000 ERCP procedures. Nurse-administered triple combination (midazolam, propofol, and pethidine) was used for sedation, and TJF-260V or JF-260V (Olympus, Tokyo, Japan) endoscope was used for examination. Furthermore, in cases with a high possibility of pancreatitis following ERCP, endoscopic retrograde pancreatic duct drainage with a stent was performed.

### Common treatment strategy following ERCP-related perforation

Intensive care including vital signs monitoring, oral intake discontinuation, intravenous broad-spectrum fluid administration, antibiotic treatment, and laboratory tests were performed when ERCP-related perforation was diagnosed. If fluid collection was suspected around the perforated area (e.g., persistent fever or leukocytosis), abdominal computed tomography (CT) was performed. If the patient complained of fever and the fluid was sufficient for drainage, percutaneous catheter drainage (PCD) was performed.

### Conventional endoscopic bile drainage treatment for ERCP-related perforation

Conventional endoscopic bile drainage was performed by ENBD and ERBD with a plastic stent. The method selection among the two conventional bile drainage methods was determined on the basis of the endoscopist’s opinion. ENBD was performed using a plastic catheter (Optimos, 7 Fr; Taewoong Medical Co., Ltd., Gyeonggi-do, Korea). Plastic ERBD was performed by placing a plastic ERBD stent over the guidewire into the bile duct. The endoscopist generally inserted a double pigtail stent (C-flex, 7 Fr and 10 cm; Boston Scientific, IN, USA) from the papilla into the left or right intrahepatic ducts. PTBD (8.5 Fr and 25 cm; Cook, IN, USA) was performed when endoscopic biliary drainage following ERCP-related perforation was not possible.

### SEMS insertion treatment for ERCP perforation

Biliary covered SEMS placement was performed along with the guidewire, which was inserted into the bile duct. The SEMS was carefully placed at the level at which the suspected perforated hole was fully covered by the metal stent. The length and diameter of the metal stent were selected on the basis of the ERCP endoscopist’s decision. There was no definite criteria for selection between conventional endoscopic and SEMS drainage for ERCP-related Stapfer type II perforations. However, when retroperitoneal fat tissue is visible through the perforated hole or sphincterotomy related bleeding accompanied, the SEMS insertion method is preferred. If the patient had no abdominal pain or fever for several days and was able to get out of gas easily, and if it was judged that placing the SEMS would no longer help the patient’s disease, the principle was to remove the SEMS before discharge. The length of stay and SEMS stay were determined by the patient’s condition. ENBD was not added after SEMS was removed.

### Other ERCP adverse events

Post-ERCP bleeding severity could be estimated by clinical significance as follows: mild (no need for transfusion, a hemoglobin decrease of <3 g/dL), moderate (transfusion of up to four units), and severe (transfusion of more than five units or need for angiographic intervention) [5]. Post-ERCP pancreatitis was defined according to the Revised Atlanta classification [6].

### Study outcomes

The perforation management success rate was the primary outcome. Secondary outcomes included the length of hospital stay, perforation-related mortality, procedure time, and other ERCP-related complications. Technical success was defined if specific management for perforation was well performed without procedure failure (e.g., successful placement of biliary stent or ENBD). Clinical success was defined if the patient with retroperitoneal perforation recovered without additional perforation managements (e.g., surgery or percutaneous abscess drainage catheter insertion) or death.

### Statistical analysis

All statistical analyses were performed using Statistical Package for the Social Sciences (version 22, IBM, Armonk, NY, USA) at a significance level of 0.05 and a confidence interval of 95%. Means of the groups were compared using Student’s t-test for normally distributed data and the Mann–Whitney *U* test for nonnormally distributed data. To compare categorical data, χ^2^ and Fisher exact tests were used. Mean (median for nonnormally distributed data), frequency, and percentage were used for descriptive analysis.

## Results

### Patients and baseline characteristics

**Fig 1:**
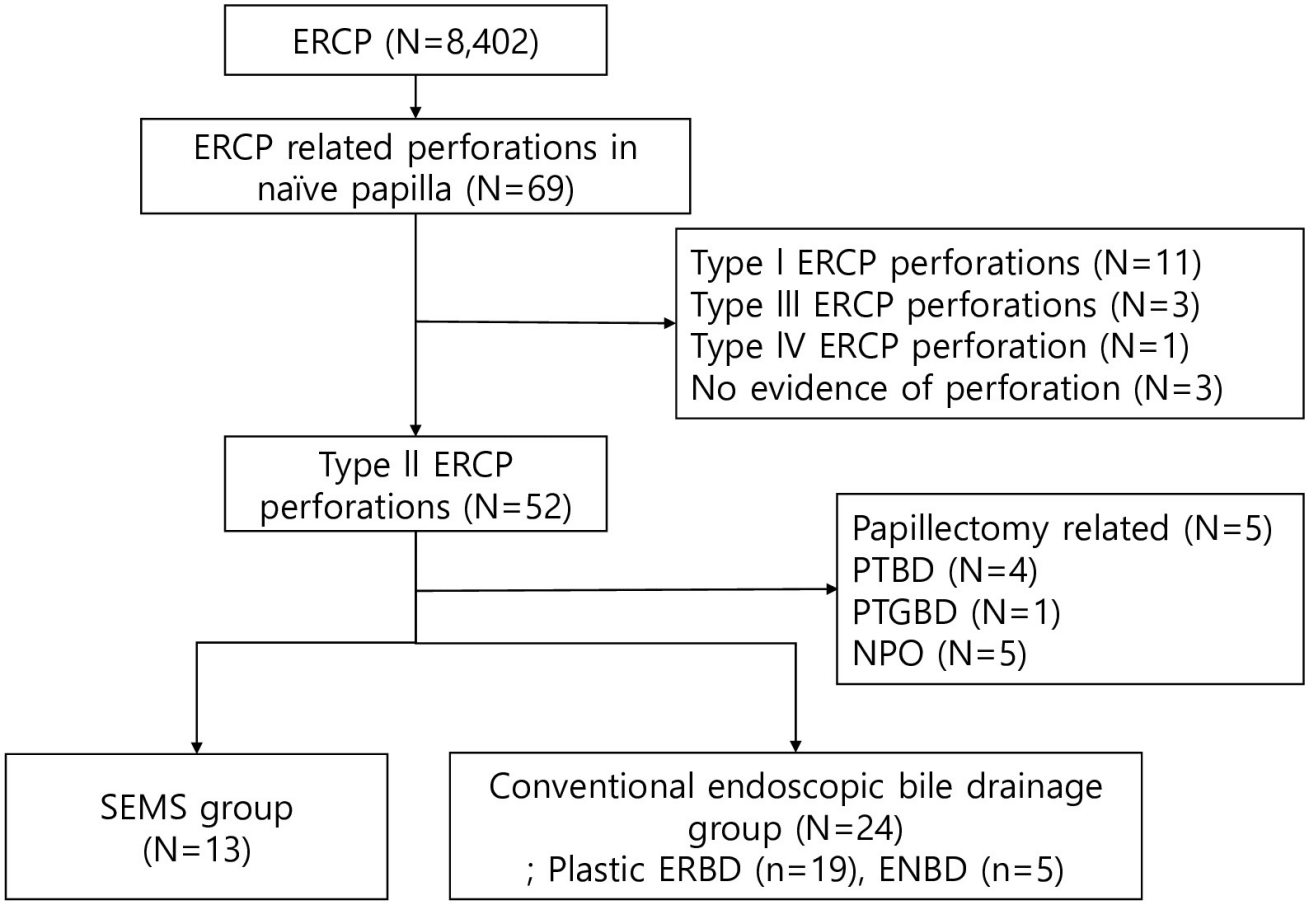
Flow chart of the study participants. ERCP, endoscopic retrograde cholanigopancreatography; SEMS, self-expandable metal stent; PTBD, percutaneous transhepatic bile drainage; PTGBD, percutaneous transhepatic gallbladder drainage; NPO, nil per os; ERBD, endoscopic retrograde biliary drainage; ENBD, endoscopic nasobiliary drainage.

A total of 8,402 ERCP procedures were performed between January 2011 and December 2022 in Kyungpook National University Hospital. These ERCP procedures were retrospectively reviewed on the basis of the prospectively described ERCP report system in our hospital. A total of 66 ERCP-related perforation cases (0.78%) were identified. Among them, after applying the exclusion criteria, 37 patients with post-ERCP Stapfer type II perforation (56.1%) were enrolled in this study. Thirteen and twenty-four patients received biliary covered SEMS insertion and conventional endoscopic bile drainage treatment, respectively (Fig 1). Except one case, 36 perforation cases were diagnosed and underwent biliary drainage during ERCP or on the day of ERCP in our study. The other one case was diagnosed and treated by plastic ERBD insertion on next day of perforation. The two groups were similar in terms of sex, ERCP indication, Charlson age–comorbidity index (CACI) [7], and baseline laboratory findings (Table 1). The conventional endoscopic bile drainage group had younger patients than the SEMS group.

**Table 1:** Characteristics of patients and baseline data.

	SEMS group (N = 13)	Conventional endoscopic bile drainage group (N = 24)	p-value
Sex, n (%) Male	6 (46.2)	13 (54.2)	0.642
Age, mean ± SD (years)	74.5 ± 12.2	64.3 ± 15.2	0.045
Charlson age–comorbidity index	5.6 ± 2.9	5.0 ± 3.0	0.526
Indication for ERCP, n (%)			
Benign biliary disease (stone/stricture/leakage/benign stricture)	10 (76.9)	17 (70.8)	1.000
Malignant biliary stricture	3 (23.1)	7 (29.2)	
Perforation cause, n (%)			
EBST	5 (38.5)	16 (66.7)	0.098
Infundibulotomy	2 (15.4)	3 (12.5)	1.000
EPLBD	1 (7.7)	2 (8.3)	1.000
Guidewire insertion	0	0	
Stone extraction	3 (23.1)	0	0.037
Forceps biopsy	2 (15.4)	2 (8.3)	0.602
Cholangioscopy	0	1 (4.2)	1.000
Perforation hole visibility in ERCP, n (%)	1 (7.7)	7 (29.2)	0.216
WBC, mean ± SD (10 e3/μL)	7,691.5 ± 3,100.4	7,439.6 ± 2,893.6	0.807
Hemoglobin, mean ± SD (g/dL)	12.3 ± 1.8	12.4 ± 1.4	0.755
Total bilirubin, mean ± SD (mg/dL)	4.5 ± 6.9	3.6 ± 5.1	0.656
Amylase, mean ± SD (U/L)	43.3 ± 30.0	114.1 ± 131.4	0.018
CRP, mean ± SD (mg/dL)	8.1 ± 10.6	2.2 ± 4.6	0.075

SD, standard deviation; ERCP, endoscopic retrograde cholangiopancreatography; SEMS, self-expandable metal stent; EBST, endoscopic biliary sphincterotomy; EPLBD, endoscopic papillary large balloon dilatation; WBC, white blood cell; CRP, C-reactive protein

### Treatment details in both groups

The most common technical cause for ERCP-related Stapfer type II perforation was endoscopic biliary sphincterotomy (56.8%), followed by infundibulotomy (13.5%), forceps biopsy (10.8%), stone extraction (8.1%), endoscopic papillary large balloon dilatation (8.1%), and cholangioscopy (2.7%). The most detailed ERCP technical causes were not significantly different between the two groups, except for stone extraction (Table 1). Nineteen (52.9%) and five (14.7%) patients underwent plastic ERBD and ENBD insertion, respectively. The type of plastic ERBD and ENBD insertion were selected on the basis of the ERCP endoscopist’s decision. ENBD insertion was usually preferred if follow-up cholangiogram or second ERCP procedure was planned. All 13 patients in the SEMS group underwent covered metallic stent with a 10-mm diameter. The length of SEMS was 56.9 ± 9.5 mm (mean ± standard deviation [SD]).

### Clinical outcomes

In both groups, the technical success rate was 100%. The clinical success rates were 92.3% (12/13 cases) and 91.7% (22/24 cases) in the SEMS and conventional endoscopic bile drainage groups, respectively, without a significant difference (p = 1.000) (Table 2). One case in each group had an exitus course. In the conventional endoscopic bile drainage group, one patient had a CACI score of 8 with a bedridden status for several years after being diagnosed with myelodysplastic syndrome. The patient visited our hospital with cholangitis due to common bile duct (CBD) stones and underwent ERCP. Stapfer type II perforation occurred during stone removal, and ENBD catheter insertion was subsequently performed. The patient recovered and was able to eat without experiencing abdominal pain and did not develop fever; however, 3 days following ERCP, he had a sudden cardiac arrest and subsequently died despite emergency cardiopulmonary resuscitation. In the SEMS group, one patient with a CACI score of 12 died of end-stage Klatskin tumor and sepsis 3 days following ERCP-related perforation. In the conventional endoscopic bile drainage group, one patient required PCD for perforation-induced abscess. One day following ERCP, the patient was diagnosed with post-ERCP Stapfer type II perforation. The patient was treated using plastic ERBD catheter insertion. However, 10 days following ERCP, the patient complained of persistent fever and abdominal pain. Abdominal CT revealed abscess formation in the right anterior pararenal space. PCD was inserted for abscess drainage. The total duration of (PC) was 61 days, and the patient recovered without any sequelae.

**Table 2:** ERCP-related clinical outcomes.

	SEMS group (N = 13)	Conventional endoscopic bile drainage group (N = 24)	p-value
Clinical success rate	12 (92.3)	22 (91.7)	1.000
Technical success rate	13 (100)	24 (100)	
Hospital days	9.4 ± 6.0	13.9 ± 13.2	0.258
NPO days	5.4 ± 3.4	4.3 ± 3.0	0.305
Complications			
Pancreatitis, n (%)	0 (0)	1 (4.2)	1.000
Fever, n (%)	4 (30.8)	6 (25.0)	0.716
Bleeding, n (%)	2 (15.4)	1 (4.2)	0.278
Death, n (%)	1 (7.7)	1 (4.2)	1.000
Total ERCP procedure time, mean ± SD (min)	18.5 ± 11.2	11.5 ± 5.2	0.013
Total hospitalization costs, mean ± SD ($)	4,624 ± 2,326	5,607 ± 5,218	0.525

ERCP, endoscopic retrograde cholanigopancreatography; SEMS, self-expandable metal stent; NPO, nil per os; SD, standard deviation

The mean hospitalization period was not significantly different between the SEMS and conventional endoscopic bile drainage groups (9.4 ± 6.0 vs. 13.9 ± 13.2 days, p = 0.258) (Table 2). Oral nutrition was established after a mean duration of 5.4 ± 3.4 versus 4.3 ± 3.0 days in the SEMS and conventional endoscopic bile drainage groups, respectively (p = 0.305). The SEMS group had a longer total ERCP procedure time than the conventional endoscopic bile drainage group (18.5 ± 11.2 vs. 11.5 ± 5.2 min, mean ± SD, p = 0.028).

### Other ERCP-related adverse events

The presence of fever and bleeding were not different between the two groups (Table 2). ERPD insertion was performed only in the conventional group (8/24 cases, 33.4%). However, there was no difference in post ERCP pancreatitis between the two groups (0 vs. 4.2%, p = 1.000).

## Discussion

In this study, conventional endoscopic bile drainage was proven as a comparative management with SEMS insertion for the treatment of type II post-ERCP–related perforations. No significant differences were observed in the clinical success rate, hospital stay and post-ERCP–related fasting time. Complications including bleeding, post-ERCP pancreatitis, fever, and death were not significantly different between the two groups. These results are likely owing to the tendency of type II perforated holes to be relatively small. Unlike Stapfer type I post-ERCP–related perforations, Stapfer type II post-ERCP–related perforations have small perforation sites. In our study, the perforation holes were only visible in 8 of 37 cases (21.6%) when we retrospectively investigated ERCP images. The exact hole size was not possible to be measured. However, all holes sizes were estimated to be at least smaller than 10 mm. Conventional bile drainage alone can reduce bile flow to the small perforated wall of the perivaterian area, thereby leading to the spontaneous healing of the perforated mucosa. Additionally, the pressure in the bile duct was decreased owing to the endoscopic biliary sphincterotomy effect. Therefore, most bile is more likely to flow toward the duodenum rather than into the small perforated hole owing to the effect of gravity and drainage tubes. However, in Stapfer type II post-ERCP–related perforations, bile sphincterotomy alone is not flawless to decrease bile pressure and allow bile flow into the duodenum. Youngelman et al. investigated whether sphincterotomy and endobiliary stenting could lower CBD pressures. Sphincterotomy alone did not decrease CBD pressure, whereas ERBD insertion with or without sphincterotomy significantly lowered CBD pressure [8].

Recent studies have demonstrated the successful treatment of Stapfer type II perforations by inserting covered metal stents instead of surgical treatment [3, 9]. In theory, the covered metal stent could block the leakage of fluid from the lesion into the retroperitoneal cavity by closing the perforated hole, thereby reducing the inflammatory response and accelerating healing. Bozbiyik et al. retrospectively compared SEMS (fully covered type, n = 19) with conventional treatment (n = 9) in type II ERCP perforation [9]. In this study, the conventional treatment included NPO, intravenous antibiotic administration, and close monitoring without other biliary drainage. This study showed that the SEMS group had better clinical results in terms of the length of hospital stay (7 [2–14] vs. 10 [7–34], days, median (range), p = 0.012). Moreover, two patients needed percutaneous drainage or surgery in the conventional treatment group, whereas no patients required percutaneous drainage or surgery in the SEMS group. Based on these results, we expected that SEMS would show superior results than conventional endoscopic bile drainage treatment in ERCP-related perforations. However, no significant difference in treatment outcomes was observed between the two groups in terms of clinical success, hospital stay, and timing of diet initiation following perforation in our study. The difference between the Bozbiyik’s and our study is biliary drainage timing. In our study, immediate endoscopic bile drainage was possible in almost all cases, except for one case. This influenced the result of two treatment modalities in our study.

Immediate bile drainage is significant to manage Stapfer type II perforations [1, 10, 11]. However, the perforation is sometimes observed following the procedure or the day following ERCP. Bozbiyik et al. investigated whether SEMS insertion time is also significant. Among 19 patients who underwent SEMS, 9 underwent biliary covered SEMS insertion during the initial ERCP, and 10 underwent biliary covered SEMS insertion in the second ERCP within 7–48 h following the initial ERCP. Either simultaneous or late SEMS insertion did not require surgical or percutaneous drainage intervention. Additionally, a significant difference in hospital stay was noted between the two groups [9]. In our study, one late plastic ERBD insertion case was noted, which was conducted the day following ERCP-related perforation. This was the only case that needed PCD for perivaterian abscess. Further well-designed studies are needed to validate SEMS effectiveness in late-noticed Stapfer type II perforations.

Among bile drainage techniques, plastic ERBD is believed to be the best option for Stapfer type II ERCP-related perforations. The indwelling drainage catheter is bothersome and uncomfortable in patients who underwent ENBD or PTBD/PTGBD. The plastic ERBD has the advantage of better compliance. Additionally, the cost of plastic ERBD is much lower than that of metallic ERBD stents [12]. In Korea, the plastic ERBD and biliary SEMS are approximately $12 and $574, respectively. However, in this study, this cost difference did not affect the total cost of hospitalizations between SEMS group and conventional endoscopic bile drainage group 4,624 ± 2,326 vs 5,607 ± 5,218, $, mean ± SD, p = 0.525). In our study, the conventional endoscopic bile drainage group had shorter procedure times than the SEMS group (11.5 ± 5.2 vs. 18.5 ± 11.2 min, mean ± SD, p = 0.013). The exact time of specific stent insertion could not be evaluated in this study owing to the limitations of a retrospective analysis. However, the baseline characteristics in indications for ERCP were not different between the two groups. Therefore, the total ERCP procedure time was believed to reflect the specific stent insertion procedure time in both groups. SEMS could still play a role in Stapfer type II ERCP-related perforations with relatively large sizes. Furthermore, we attempted to determine the exact size of the hole in perivaterian perforations. Among the 37 cases, perforation holes were seen retrospectively in 8 cases, 7 of which were in the conventional endoscopic bile drainage group. Although, all holes sizes were estimated to be at least smaller than 10 mm, we could not obtain the exact size of the perforated hole despite reviewing the endoscopic images in all ERCP study patients owing to the limitations of a retrospective analysis. Further, prospective studies are warranted to validate SEMS in Stapfer type II ERCP-related perforations according to perforated hole size.

Of the four types of ERCP-related perforations, Stapfer type II perforation occurring in the perivaterian or peri-ampulla is usually caused by sphincterotomy or precut papillotomy. In our study, when excepting small number of papillectomy cases and including external bile drainage/NPO cases, Stapfer type II perforation was the most common type and accounted for 71.2% (47/66 cases) of ERCP-related perforations, which is consistent with those of other studies [1, 2]. Furthermore, endobiliary sphincterotomy (44.7%) was the most common cause of Stapfer type II perforation. ERCP type I perforation usually requires surgical management. In contrast, Stapfer type II perforations can be mostly treated by nonsurgical methods [3]. However, Assalia et al. reported that immediate surgical management could be required in some patients with Stapfer type II perforation. Additionally, death was reported in one patient who had severe comorbidities and underwent nonsurgical management [10]. In the current study, most Stapfer type II ERCP-related perforation cases (34/37 cases, 91.9%) were successfully treated by nonsurgical treatment, except for three cases. Of three cases, two had severe underlying diseases, including hematologic and biliary malignancy, and eventually led to death. The other case had a relatively larger amount of perforation-related perivaterian fluid leakage requiring external drainage. The patient finally recovered without other complications. However, the patient had to keep the external drainage catheter for over 3 months. In the case when surgical management is required following failure of nonsurgical management, delayed time to operation is a significant factor that determines prognosis. Therefore, treatment modality selection is challenging in post-ERCP perforation [13, 14]. In Stapfer type II ERCP-related perforation, a multidisciplinary approach that includes a radiologist, surgeon, and an endoscopist is necessary even if the initial management is planned on the basis of nonsurgical management.

Post ERCP pancreatitis is another fatal complication associated with ERCP. In particular, if accompanied by perforation, the prognosis is expected to be worse. Previous studies have reported that the prevalence of post ERCP pancreatitis in cases of post ERCP perforation is 10–43% [3, 9, 15]. Compared to previous studies, this study confirmed that the prevalence of post ERCP pancreatitis was very low at 1 in 37 cases (2.7%). The ERPD intubation rate in our study was at 33.4%. The cause is believed to be that pancreatitis pain may be masked due to perforation and that the number of cases in this study was not large. Additional research is needed in the future regarding the role of ERPD insertion in preventing pancreatitis accompanying post ERCP perforation.

This study had some imitations. First, it had a retrospective design. As our center had a prospective ERCP database report recording system, ERCP perforation rates and types were believed to be similar to real clinical situations. However, we could not identify the details of procedure-related minor complications and the reason for specific management selection. Second, this study had a small sample size. As the post-ERCP perforation rate is very low, a further multicenter study design is warranted to validate our results. Third, only immediately diagnosed perforations were included in this study. Delayed perforation was not accessed. However, previous studies have shown that the early detection rate of ERCP-related perforation is approximately 78% [1, 16]. Therefore, early diagnosed ERCP-related perforations, as in our study, can be considered representative of most ERCP-related perforation cases.

## Conclusions

The conventional endoscopic bile duct drainage method showed similar effective clinical outcomes and a shorter procedure time than biliary metal stent insertion in ERCP-related Stapfer type II perforations.
